# Letters on Quackery and Answers to Correspondents

**Published:** 1872-04

**Authors:** 


### LETTERS EXPOSING HUMBUGS AND SWINDLERS, WITH OTHER COMMUNICATIONS, OF INTEREST TO THE PEOPLE.

PHILADELPHIA Jan 17th 1872

DR UP DEGRAFF

*Sir* My attention was called to an article in your January issue the whole issue is a piece of imposition practiced on you I never wrote it Yours A P BISSELL
914 Lombard-St

The above effusion is in reply to a letter published in the January number of the BISTOURY, purporting to have been written by the gentleman whose name appears above, offering the sale of a medical diploma. *Perhaps* he did not write the letter, but the circumstance of its having been penned upon paper bearing the letter-head, and advertisement of the "Eclectic Medical College of Penn.," and the further fact, that we are in possession of abundant evidence that the gentleman named, *has* sold diplomas of the College which he represents, renders it possible, if not quite probable, that he is the author of the letter referred to.

We are glad to see that the Legislature of Pennsylvania has appointed a committee to examine these cheap medical colleges, with which Philadelphia has been so long disgraced; and that the diploma-selling concerns are likely to come to grief.

LATER.—The telegraph has just announced, that the charters of the "Eclectic Medical College," and "The Phila. University of Medicine," have been canceled by the Senate of Pennsylvania. "Good enough."

---

SHAMOKIN, Pa., March 9th, 1872.

DR. UP DE GRAFF:

*Dear Sir:*—What do you know of Dr. O. Phelps Brown, who advertises in all the magazines of the country, to cure Epilepsy or Fits? Is he to be trusted? L——.

It is safe to say that he is an impostor. Physicians who are skilled in any branch of medical science do not require to advertise themselves into notoriety. Better employ your regular family physician—even if he is an "old fogy," or as you suppose, "not posted," he is ten times better and safer than *any* advertising quack.

---

JAMESTOWN, N. Y., March 11, 1872.

DEAR BISTOURY:

You seem to be posted upon all subjects, and give so much useful information, that I thought perhaps, you might tell me what to do for a valuable horse, which is frequently troubled with colic. If you can suggest anything for his relief you will greatly oblige,

AN OLD SUBSCRIBER.

Now, that's just in our line.—We have a pony that, a short time since, was seriously ill, with what was supposed to be, colic.—Not being "posted" in horse ailments, we sent for a "Veterinary Surgeon." He came, felt the horse's pulse, and pronounced him to be suffering from colic. He left directions and medicines, and the pony recovered. This little experience cost us just $20, the sum charged us by the Veterinary Surgeon. Inasmuch as he would not take the pony in part pay, of course, we paid the bill. Now, therefore, if your horse is subject to colic, kill him; it will be much cheaper, provided *your* Veterinary chaps charge as ours do.

But since we have related the symptoms of our pony to others, we have discovered more than a thousand cures for colic;—they will all cure, and are sure fire. They consist of everything, or anything,—you can't go amiss. Give him boiling water, hot shot, salt, saleratus, soap, (hard or soft, makes no difference), matches, bricks, (hot or cold), potatoes, alum, ashes, tobacco, (fine cut, or plug). Bleed him in the mouth, pour melted butter in his ears, place a hot-onion-poultice over his abdomen, smear him all over with mustard, pull him in a continual circle by the tail, (hire out this last job to some fellow anxious for exercise, as you will find it fatiguing after the first hour), rub salt on his back, with a scrubbing brush, punch his belly with a mop; if bloated, like a bondholder, thrust your jack-knife into his belly,—need not be particular about the spot, one place is equally as good as another,—fill a clay pipe with tobacco, light it, and use the stem as you would a suppository; it is said you can convert your horse into a cheerful smoker in this manner, and greatly relieve the monotony of his position, if not the colic. These are a few of the *actual* remedies that were suggested to us by farmers and jockeys, skilled in the management of horse-flesh. Every one of them are genuine, and were pronounced by their respective advocates to be "sure cures." We hope you will experience no serious difficulty in selecting your cure from the above list, it certainly is varied enough, but we cannot hold ourself responsible for the results you may obtain in the use of *any* of them. And this is "all we know about colic in horses."

---

WILLIAMSPORT, Pa., Feb. 20, 1870.

DR. UP DEGRAFF:

I have a large wart upon the back of my hand. What can I do to get rid of it?

L——.

Procure two grains of *chromic acid*, in a drachm bottle. Allow the cork to remain out the bottle over night. In the morning the acid will have absorbed sufficient moisture to be liquid. Take a small pine stick, place it in the acid, and smear the wart thoroughly with the remedy. Do not wet the hand after the application. Apply the acid morning and evening, for from five to ten days. You will have the satisfaction of seeing the wart detach itself and come out entire. Smear a cloth with fresh lard, and keep over the hole left by the wart, until it heals.

---

*H. L. M.—Cohoes, N. Y.*—Your doctor is right. A felon should be opened to the bone, as soon as you discover it to be one. You should *not* wait until matter forms. Open immediately, then poultice.

---

*Dr. R.—Memphis, Tenn.*—Flint on Diseases of the Heart. Wells on diseases of the eye.

---

*L. U.—Williamsport, Pa.*—It is *now* after the holidays!

---

*H.—Selinsgrove, Pa.*—No safe treatment can be laid down for the cure of enlarged tonsils with ulceration of the throat, which you can conduct yourself. Better take your child to your family physician for treatment. The tonsils, perhaps, require removing.

---

*B.—Watsontown, Pa.*—We have repeatedly notified our readers that we do not travel, neither do we countenance traveling doctors of any description. The man bearing our name, of whom you write, is in no manner connected with us, or our institution.

*Mrs. M.—Salem, O.*—We have answered your question more than one dozen times in the BISTOURY. Never use a cancer plaster of any description,—never employ a "cancer doctor,"—never squander your money on a traveling doctor.

---

*Dr. S.—Rochester, N. Y.*—You can procure a galvano-cautery battery, that will answer your purpose, for about $40. Write Geo. Tiemann & Co., N. Y.

---

ROCHESTER, Ind., Feb. 15, 1872.

DEAR SIR:

As your paper is especially devoted to the exposure of empiricism, I send you the advertisement of Dr. Storz, the self-styled "celebrated Lung physician, late of Philadelphia, now of Chicago," who represents himself as a "graduate of the Eclectic Medical University of Philadelphia, and the (old school) Penn. University of Med. & Surgery, and Physician to the Philadelphia Charity Hospital, Surgeon in the Army," &c., &c. How is that for high?

Now what are the facts? There is no school of medicine in Philadelphia styled the Eclectic Medical University, and never was. There is no Hospital styled the Phila. Charity Hospital, and never was. There was a nondescript mixed school for boys and girls, styled the "Penn. University," which died 14 years ago of consumption, and which was never recognized by the profession as a regular or "*old* school" institution. So all the Doctor's assumptions are false, and gotten up to deceive. He has no diploma from any recognized School, regular or irregular, unless one of the two quack schools of Philadelphia, who *sell diplomas*, without medical tuition, have furnished him one since his bills were issued, —*for a consideration.*

The celebrated Lung Physician was pretty well ventilated this winter by the Rennselaer *Union*, Winamac *Democrat*, and Francesville *Topic*, and he threatened to *persecute* any one who dared to *attact* his medical *repetation*!

There is a *perscription* in one of our stores from him for a pint of "wrhye whiskey," and "golden seel," but whether the medicine "fotched the tape worm" or not, is unknown.

Side by side with Dr. Storzs' bills, in one of our hotels, stands the following in MSS.:

"The renound DoCtor Collins wile be at the undersined hotell on Oct 12th He cures Dispepsy and all Kronick Diseases such as eye and ear complaints, Neuralgy, and all diseases without medicine. Come and see him no cure no pa Austin House."

Another: Dr. Sandford the celebrated tape worm physician of Logansport, offers a thousand dollars for every case of Fits he can't cure. For 50c. he proposed to remove the discoloration of an epileptic, who had been treated with large doses of nitras argentum.—He stated to me that she had been greatly maltreated by the use of *quicksilver!* The ass really did not know the difference between nitrate of silver and mercury. The old farmer, however, did not *take*, and the fits remain and the discoloration also.

Another: Dr. Bort, of Ft. Wayne, pasted up posters over our fences, with letters a foot high: "The Great Dr. Bort is Coming," and advertises, (occupying one whole page of our county papers), cases that never occurred, at places that never had an existence. One lady in Fulton county, (where I now write), "blind from birth, for 26 years, he restored to perfect vision in 24 hours."

Such asses are thicker than the locusts of Egypt, out west. They are on every railroad, stop at all stations, humbug the people, steal their money, and are always ready for the next one that comes along. Well for the ignoramuses, that patronize these scamps, if they lost only their money; but many of them lose both money and health irretrievably. I know of no remedy but exposing their pretensions, till the Legislature shall be induced to pass laws making it a *felony* to practice without a diploma from some recognized and responsible institution, and repealing the charters of all such as sell their diplomas, as is charged and well proved against the two quack schools of Philadelphia. See Philadelphia Press of May 1st, '71, and the Bulletin of Nov.—same year. Yours, truly, D. L. T.

